# From Saliva to Diagnosis: A Scoping Review of Conventional and Biosensor-Based Methods for Salivary Biomarkers in Chronic Kidney Disease

**DOI:** 10.3390/diagnostics15172226

**Published:** 2025-09-02

**Authors:** Elena Valentina Vacarel, Eliza Denisa Barbulescu (Sgiea), Corina Marilena Cristache

**Affiliations:** 1 Doctoral School, “Carol Davila” University of Medicine and Pharmacy, 37 Dionisie Lupu Street, 020021 Bucharest, Romania; elena-valentina.vacarel@drd.umfcd.ro (E.V.V.); eliza-denisa.sgiea@drd.umfcd.ro (E.D.B.); 2 Department of Dental Techniques, “Carol Davila” University of Medicine and Pharmacy, 8 Eroii Sanitari Blvd., 050474 Bucharest, Romania

**Keywords:** salivary biomarkers, chronic kidney disease, non-invasive diagnostics, biosensors, point-of-care testing, dental screening, diagnostic accuracy

## Abstract

Background: Chronic kidney disease (CKD) is a progressive global health burden often diagnosed in late stages due to reliance on invasive and centralized blood and urine tests. Saliva, as a non-invasive diagnostic fluid, has emerged as a promising alternative for assessing renal function. This scoping review aims to evaluate the diagnostic accuracy of salivary biomarkers compared to traditional methods, and to explore the potential of emerging biosensing technologies for CKD detection and monitoring. Methods: A comprehensive literature search was conducted in PubMed/MEDLINE, Scopus, Web of Science, and Cochrane Library up to 1 July 2025, following the PRISMA-ScR guidelines. Studies involving adult CKD patients and healthy controls that assessed the diagnostic performance of salivary biomarkers against validated reference standards (e.g., serum creatinine, eGFR) were included. A total of 29 eligible studies were selected after applying predefined inclusion and exclusion criteria. Results: Salivary creatinine and urea were the most frequently assessed biomarkers and demonstrated strong correlations with serum levels (AUCs up to 1.00; sensitivity and specificity frequently >85%). Several studies reported high diagnostic potential for novel salivary markers such as Trimethylamine N-oxide (TMAO), cystatin C, and amino acids. Technological innovations, including electrochemical biosensors and ATR-FTIR spectroscopy, showed promise for enhancing sensitivity and enabling point-of-care testing. However, heterogeneity in sampling protocols and limited data for early-stage CKD were notable limitations. Conclusions: Salivary diagnostics, supported by biosensor technologies, offer a feasible and non-invasive alternative for CKD screening and monitoring. Standardization, broader clinical validation, and integration into dental workflows are key to clinical implementation.

## 1. Introduction

Chronic kidney disease (CKD) is a progressive and irreversible condition marked by structural or functional abnormalities of the kidney lasting for more than three months [1]. Affecting over 850 million people globally—approximately 8–16% of the world’s population—CKD is a major public health challenge with a silent clinical course in its early stages [2,3]. Diagnosis often occurs only when significant renal impairment has already developed, limiting the effectiveness of early interventions. The disease is staged based on estimated glomerular filtration rate (eGFR) and albumin-to-creatinine ratio (ACR), as recommended by the Kidney Disease: Improving Global Outcomes (KDIGO) guidelines [1,3].

Diabetes mellitus, particularly type 2, and hypertension are the leading causes of CKD, although a considerable number of cases remain of unknown etiology [4]. Without timely diagnosis and management, CKD progresses toward end-stage kidney disease (ESKD), requiring dialysis or kidney transplantation and contributing to high morbidity, mortality, and healthcare costs. By 2030, the number of individuals requiring renal replacement therapy is projected to reach 5.4 million, and by 2040, CKD is expected to be the fifth leading cause of death worldwide [5].

Historically, serum creatinine and urea have been the primary markers for assessing renal function, with blood sampling considered the gold standard [6,7]. While these methods are clinically validated and widely used, they are invasive, time-consuming, and dependent on laboratory infrastructure [8]. This imposes logistical and economic burdens, particularly in low-resource settings, and can negatively impact patient compliance with regular monitoring [8,9].

In this context, saliva has emerged as a promising noninvasive alternative for assessing renal function [8,10,11]. Saliva offers several practical advantages: it is easy to collect, minimally invasive, painless, and does not require specialized personnel or equipment, making it especially attractive for point-of-care and home-based testing. Saliva is considered a filtrate of blood, reflecting systemic biochemical changes through transcellular and paracellular transport pathways [12]. Consequently, biomarkers commonly used to assess kidney function—such as creatinine, urea, cystatin C, and various electrolytes—have been detected in saliva with varying degrees of correlation to serum and urine levels [13,14,15].

Recent studies have reported encouraging evidence of the diagnostic potential of salivary biomarkers, with some demonstrating strong correlations with their serum counterparts, particularly for creatinine and urea [14,16]. However, there remains considerable variability in results across CKD stages and patient populations, particularly in early disease detection and post-dialysis monitoring [17]. This inconsistency presents a significant knowledge gap regarding the diagnostic validity, sensitivity, and specificity of saliva-based assessments. Without a clearer understanding of these parameters, the widespread clinical adoption of salivary diagnostics remains limited.

The present scoping review addresses this gap by evaluating both traditional and emerging approaches to CKD biomarker detection, with a special focus on salivary diagnostics. It explores the integration of conventional biochemical assessments with advancements in biosensing technologies—such as electrochemical sensors, immunoassays, and microfluidic platforms—that aim to overcome current limitations in sensitivity and analytical reliability. These biosensor-driven strategies offer potential for rapid, low-cost, and decentralized diagnostics, which could transform CKD screening and monitoring, particularly in underserved populations.

By examining the diagnostic equivalence and clinical utility of salivary biomarkers compared to conventional fluids, this review aims to provide a comprehensive overview of the current evidence and guide future research in the development of accessible, noninvasive diagnostic tools for chronic kidney disease.

Saliva-based diagnostics address critical gaps in CKD care by offering a painless, rapid, and potentially home-based alternative to current invasive methods. While individual biomarkers such as creatinine and urea have been repeatedly validated, variability in methodologies, limited early-stage detection accuracy, and scarce integration of emerging biosensor technologies hinder clinical adoption. This review responds to these gaps by mapping conventional and novel salivary biomarkers alongside biosensing approaches, thereby providing an updated and multidisciplinary perspective to inform research, regulation, and clinical implementation.

## 2. Materials and Methods

This scoping review explored existing studies assessing the diagnostic performance, specifically the accuracy, sensitivity, and specificity, of salivary biomarkers in detecting CKD. It also aimed to identify existing limitations in terms of standardization, clinical implementation, and technological innovation. A comprehensive literature search was carried out across multiple databases, including PubMed/MEDLINE, Scopus, Web of Science, and the Cochrane Library, without applying any publication year filters, up to 1st of July 2025. The search strategy followed the Preferred Reporting Items for Systematic Reviews and Meta-Analyses for scoping reviews (PRISMA-ScR) guidelines [18] and was registered with the Open Science Framework (https://doi.org/10.17605/OSF.IO/BHKRX accessed on 27 August 2025). To ensure completeness, supplementary searches were conducted in gray literature sources, specialized Google search tools, relevant institutional or scientific websites, and the reference and citation lists of the selected publications. The research question was formulated using the PICOS framework (Population, Intervention, Comparator, Outcome, Study Design), as outlined in Table 1. The search utilized a combination of Medical Subject Headings (MeSH), keywords, synonyms, and free-text terms, combined with the Boolean operators “AND” and “OR”. The complete search strategy and the PRISMA-ScR checklist can be found in the link: https://doi.org/10.17605/OSF.IO/BHKRX (accessed on 27 August 2025).

The review was guided by two central questions: (1) How do salivary biomarkers (e.g., urea, creatinine, ammonia, pH) compare to traditional blood and urine-based diagnostics in terms of accuracy, sensitivity, and specificity? (2) How can digital salivary diagnostic tools be incorporated into dental clinical workflows for early CKD screening and monitoring of systemic health?

The specific objectives of this review were:

- To evaluate the diagnostic accuracy, sensitivity, and specificity of salivary biomarkers in CKD.
- To compare salivary biomarkers with traditional blood and urine markers for CKD diagnosis and monitoring.
- To assess which salivary biomarkers demonstrate the highest diagnostic performance and are most suitable for guiding dietary management, continuous monitoring, and referral for medical intervention or dialysis.
- To explore how oral health factors and dental clinical workflows influence the reliability and integration of salivary diagnostics for CKD detection.
- To assess the technologies and methodologies used to detect CKD-related biomarkers in saliva, including biosensors, spectrophotometry, and microfluidic devices.
- To identify limitations and challenges in the clinical application of salivary diagnostics for CKD.
- To propose future directions and standardization strategies for the implementation of saliva-based diagnostics.

### 2.1. Outcome Measures

The primary outcome measure was the diagnostic performance of salivary biomarkers for CKD, expressed as correlation with serum measures, area under the receiver operating characteristic curve (AUC), sensitivity, and specificity. Secondary outcomes included the identification of emerging salivary biomarkers, the description of biosensor-based analytical methods, and the reported feasibility of clinical integration, particularly in dental settings.

### 2.2. Eligibility Criteria

Studies were eligible for inclusion if they involved adult participants (≥18 years) with a confirmed diagnosis of CKD or at risk for CKD (e.g., due to diabetes or hypertension) and reported on salivary biomarkers relevant to kidney function. Eligible studies evaluated diagnostic accuracy metrics (e.g., sensitivity, specificity, Area Under the Curve -AUC) or reported correlations between salivary and serum or urine biomarker levels. Only studies comparing salivary findings with traditional blood or urine markers were considered. Additional inclusion criteria required a minimum of 20 CKD patients and the inclusion of a healthy control group. Furthermore, studies were required to use a validated reference standard for CKD diagnosis, such as glomerular filtration rate (GFR), serum creatinine (sCr), or blood urea nitrogen (BUN).

Excluded were pediatric studies, preclinical (animal or in vitro) investigations, and studies involving acute kidney injury or conditions known to severely alter salivary composition (e.g., Sjögren’s syndrome, salivary gland irradiation). To avoid redundancy with prior systematic reviews, studies focusing solely on changes in salivary biomarkers before and after hemodialysis in ESKD patients with or without a comparator healthy group were also excluded. This decision was made in light of the systematic review with meta-analysis by Rodrigues et al., which thoroughly evaluated this specific clinical context [7].

Eligible study designs included observational studies (cross-sectional, cohort, case–control), diagnostic accuracy studies, and clinical validation studies. Reviews, editorials, case reports, and non-comparative studies were excluded.

After removing duplicates, title and abstract screening was conducted in a blinded manner using Catchii.org (accessed on 27 August 2025) by two independent reviewers (E.V.V. and E.D.B.), followed by full-text assessment of the eligible articles. Discrepancies were resolved through discussion or by a third reviewer (C.M.C.). Data were extracted using a structured Excel form capturing study characteristics, population details, salivary biomarkers assessed, comparator tests, analytical methods, diagnostic outcomes, and technological readiness.

## 3. Results

The initial search across the four databases yielded a total of 753 publications. After screening, 219 duplicates were removed, and 516 studies were independently assessed by the two reviewers based on title and abstract. A total of 449 studies were excluded during this stage. The full texts of the remaining 67 studies were retrieved and evaluated against the inclusion and exclusion criteria.

Out of these, 50 studies were excluded for the following reasons:

- 2 were poster abstracts presented at the 49th Turkish Physiology Congress of the Turkish Society of Physiological Sciences in 2024 [19],
- 15 were in vitro studies [16,20,21,22,23,24,25,26,27,28,29,30,31,32,33],
- 14 were review articles [7,8,34,35,36,37,38,39,40,41,42,43,44,45], and
- 19 for not meeting the inclusion/exclusion criteria (e.g., sample size fewer than 20 participants [46,47], absence of specific CKD patient cohorts [48], lack of a healthy comparator group [12,13,14,17,49,50,51,52,53,54,55,56,57], lack of validated kidney function assessment methods [58,59], or inclusion of pediatric populations [60]).

Meanwhile, an additional search in other databases yielded 17 more records. After eligibility assessment, 12 of these were included, resulting in a total of 29 studies [2,3,5,9,61,62,63,64,65,66,67,68,69,70,71,72,73,74,75,76,77,78,79,80,81,82,83,84,85] included in the scoping review.

**Figure 1:**
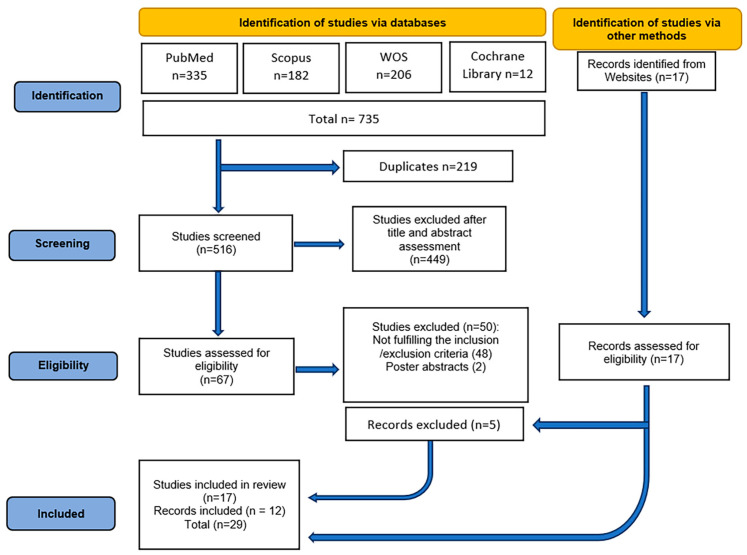
PRISMA [86] flow diagram showing the number of records identified, included and excluded.

The PRISMA flow diagram illustrating the study selection process is presented in Figure 1.

Table 2 summarizes the key data extracted from the 29 included studies. The table presents information on study design, population characteristics, biomarker(s) investigated, methods of saliva collection and analysis, and main findings.

**Figure 2:**
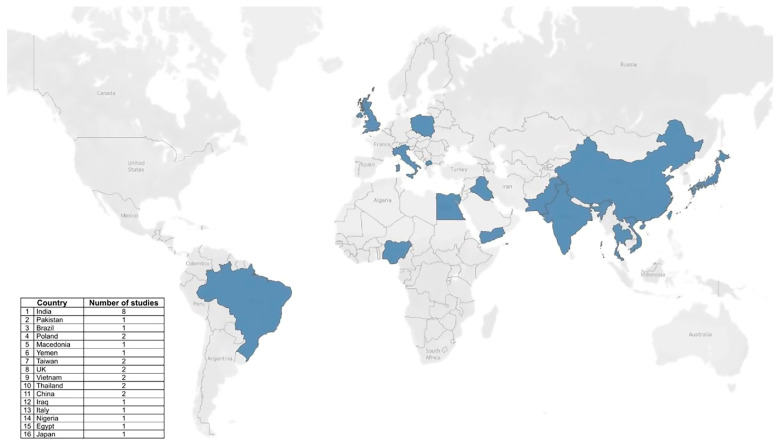
Geographic distribution of the included studies.

Across the 29 included studies, sample sizes ranged from 20 to 214 participants, with 6 studies exclusively investigating advanced CKD (stages 4–5 and/or ESKD), 19 enrolling mixed-stage CKD cohorts, and 4 specifically including participants with associated systemic conditions such as diabetes mellitus or diabetic nephropathy. Geographic representation was broad (Figure 2): Asia (*n* = 18 studies; 8 from India, 2 from China, 2 from Taiwan, 2 from Thailand, 2 from Vietnam, 1 from Japan, 1 from Pakistan), Europe (*n* = 6; Poland, UK, Italy, North Macedonia), the Middle East (*n* = 3; Iraq, Yemen, Egypt), South America (*n* = 1; Brazil), and Africa (*n* = 1; Nigeria). Study designs included 12 cross-sectional, 7 observational, 5 case–control, and 5 diagnostic accuracy/validation studies. Most were conducted in university or hospital settings, with dental hospitals contributing to four studies. CKD diagnosis was confirmed in all cases using validated reference standards such as serum creatinine, eGFR, or blood urea nitrogen.

Regarding the biomarkers tested, creatinine and urea remain the most validated salivary markers, showing strong correlations with serum levels and CKD stage. The diagnostic performance of salivary creatinine and urea across studies is summarized in Table 3.

In addition to conventional well tested biomarkers, creatinine and urea, several studies explored novel salivary markers such as TMAO, cystatin variants, specific amino acids, and proteomic profiles. Table 4 summarizes these exploratory findings, which may contribute to improved non-invasive diagnostics pending further clinical validation.

## 4. Discussion

CKD represents a major global health burden, affecting millions of individuals and contributing to over 1.4 million deaths and more than 40 million disability-adjusted life years annually [1]. The increasing prevalence of CKD is largely driven by aging populations and the widespread incidence of hypertension and diabetes [87]. Current diagnostic practices rely heavily on serum and urine analyses, such as eGFR and albumin-to-creatinine ratios, which, while effective, often present logistical and economic challenges—particularly in resource-limited settings [88]. Despite advancements in clinical protocols, there remains a critical unmet need for non-invasive, accessible, and cost-effective diagnostic tools that can facilitate early detection and continuous monitoring of CKD [89]. Developing alternative approaches that overcome the limitations of traditional testing is essential for improving clinical outcomes and reducing the overall burden of this chronic condition [90].

Previous reviews on salivary biomarkers for CKD have primarily focused on well-established analytes such as creatinine and urea, with limited exploration of emerging molecular candidates and biosensing technologies. For instance, Celec et al. summarized biochemical changes in saliva associated with systemic diseases, including CKD, but without detailed diagnostic accuracy metrics [38]. Rodrigues et al. (2020) and Rodrigues et al. (2021) reviewed salivary creatinine and urea correlations with serum measures, yet did not extensively address newer biomarkers or the potential of point-of-care devices [6,7].

Our review advances current knowledge by: including the most recent evidence up to July 2025 across four major databases and grey literature; systematically mapping both established and emerging biomarkers with diagnostic potential, including cystatin C, TMAO, and targeted amino acid panels; assessing novel biosensing technologies such as electrochemical platforms and ATR-FTIR spectroscopy, with emphasis on their potential integration into dental care workflows; and identifying persistent gaps in early-stage CKD detection, standardization of saliva collection/analysis, and the need for health economic evaluations. This multidisciplinary perspective is intended to support translational research and the adoption of saliva-based diagnostics in both medical and dental clinical practice.

Saliva is increasingly recognized as a valuable biological fluid for non-invasive diagnostics, offering a practical alternative to blood and urine testing, particularly in point-of-care settings [37,91]. Its collection is simple, safe, and well tolerated, making it suitable for populations where venipuncture may be challenging, such as pediatric, elderly, or chronically ill patients [1]. The diagnostic potential of saliva is rooted in its rich and dynamic composition, which mirrors many of the biomolecules present in systemic circulation [91].

Physiologically, saliva is a viscoelastic and hypotonic fluid secreted primarily by the parotid, submandibular, and sublingual glands. Its production is tightly regulated by the autonomic nervous system, with parasympathetic stimulation favoring serous secretion and sympathetic activity enhancing mucous output [92]. On average, adults produce between 500 and 1500 mL of saliva daily, and its flow rate and composition are influenced by circadian rhythms, gland type, and external stimuli [93]. Saliva transitions from an isotonic fluid at the acinar level to a hypotonic one in the ducts due to ionic modifications, particularly the reabsorption of sodium and chloride [94].

The biochemical complexity of saliva is remarkable, with over 2000 proteins and peptides identified, including enzymes (e.g., α-amylase), mucins, antimicrobial peptides, hormones, and immunoglobulins. It also contains electrolytes such as sodium, potassium, calcium, and bicarbonate, the latter contributing significantly to its buffering capacity and maintenance of oral pH [95,96]. Many salivary components—such as cortisol, creatinine, urea, and albumin—have diagnostic relevance, as their concentrations reflect systemic physiological and pathological states. In fact, around 27% of salivary proteins are shared with blood, supporting the feasibility of saliva-based diagnostics for systemic diseases [37].

Beyond composition, the mechanisms facilitating the entry of systemic biomarkers into saliva further enhance its diagnostic value. Molecules reach saliva through transcellular diffusion, active transport, or paracellular ultrafiltration via salivary acini and gingival crevices [97,98]. This enables the detection of a broad range of analytes, including low-molecular-weight substances like urea and creatinine, which are particularly relevant in renal disease monitoring. Additionally, hormones and other small lipophilic molecules diffuse readily into saliva, allowing for hormonal profiling and stress assessment [99].

As a historical overview, salivary biomarker research in CKD has evolved significantly over the past four decades. Pioneering studies in the 1980s laid the foundation, Akai et al. (1983) [80], for instance, demonstrated that salivary urea nitrogen levels closely reflected serum values using a urease-based dry reagent strip read by a reflectance spectrometer, achieving a high correlation coefficient (r ≈ 0.93).

By the mid-1990s, researchers like Lloyd et al. (1996) [65] extended this concept to salivary creatinine, showing that creatinine concentration in saliva (about 10–15% of the serum level in healthy individuals) rises dramatically in CKD patients and correlates strongly with impaired renal function. Lloyd’s clinical validation achieved nearly 100% sensitivity and ~96% specificity for detecting elevated serum creatinine using a saliva cutoff, solidifying saliva’s potential as a noninvasive diagnostic fluid. In subsequent years, key methodological shifts enhanced the reliability of salivary tests: collection techniques became more standardized (e.g., fasting morning samples or swab-based collection to ensure consistency), and analytical technologies advanced from simple colorimetric assays to sophisticated platforms.

Today, portable biosensors enable rapid, on-site measurement of salivary biomarkers with impressive sensitivity. Nanomaterial-based electrochemical sensors, for example, can detect creatinine at concentrations far lower than those measurable by traditional Jaffé assays, addressing earlier limitations in detection thresholds. Spectroscopic techniques such as attenuated total reflectance FTIR (ATR-FTIR) [3] spectroscopy have also been introduced, allowing for non-reagent-based quantification of salivary urea and detection of broader biochemical signatures associated with uremia. In parallel, there has been a notable shift from single-analyte approaches toward multi-marker panels and omics-based strategies. Recent studies have demonstrated the feasibility of simultaneously measuring urea, creatinine, cystatin C, and additional metabolites to improve diagnostic precision and staging accuracy in CKD.

**Figure 3:**
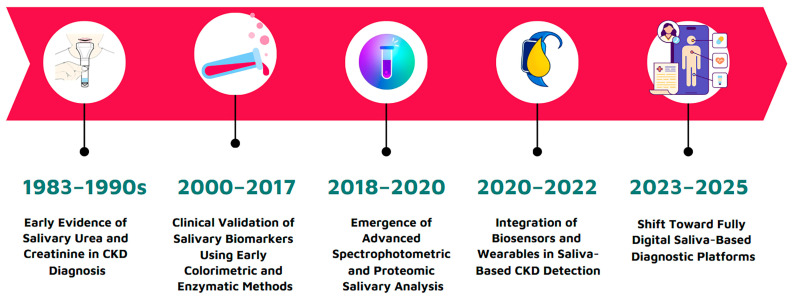
Historical progression of salivary diagnostics in nephrology, highlighting key milestones from the early validation of urea and creatinine to the integration of biosensing, spectroscopy, and multiplex assays.

This historical progression, from early validations of urea and creatinine to the integration of biosensing, spectroscopy, and multiplex assays, underscores the growing analytical robustness and clinical relevance of salivary diagnostics in nephrology. An overview of key milestones and validation data is illustrated in Figure 3.

Following these technological and methodological advances, numerous contemporary studies have confirmed the high diagnostic accuracy of salivary biomarkers in CKD. For example, Padwal et al. [67] and Venkatapathy et al. [75] both reported AUCs exceeding 0.95 for salivary creatinine, with sensitivities and specificities approaching 100%. Likewise, Choudhry et al. [61] found strong diagnostic performance for both urea (AUC: 0.78) and creatinine (AUC: 0.86). Supporting these results, Tangwanichgapong et al. [3] employed ATR-FTIR spectroscopy and achieved near-perfect sensitivity and specificity (100%) in their comparisons. Overall, sensitivity values for salivary biomarkers have ranged between 75% and 100%, while specificity has typically fallen between 80% and 100%, confirming their reliability for detecting CKD—particularly in its more advanced stages.

In addition to these diagnostic performance metrics, a strong and consistent correlation between salivary and serum levels of urea and creatinine has been observed across multiple studies. Lasisi et al. [9], Bagalad et al. [84], and Pham [76] all reported high correlation coefficients, indicating that salivary levels closely track serum elevations. Notably, Khursheed et al. [63] found that an electrochemical biosensor provided superior salivary creatinine recovery compared to the conventional Jaffe method. However, despite these encouraging findings, other studies such as Picolo et al. [2] and Wang et al. [77] suggest that integrating salivary biomarkers with systemic parameters, like age or diabetes status, may enhance predictive accuracy, particularly in early-stage CKD.

Among the investigated biomarkers, creatinine and urea remain the most validated and widely studied, consistently demonstrating robust performance. In addition, emerging markers offer potential for more refined monitoring and staging. For example, TMAO (trimethylamine N-oxide), as reported by Korytowska-Przybylska et al. [64], may help distinguish CKD stages and guide dietary interventions. Similarly, cystatin C and specific amino acids (e.g., arginine, valine, histidine, as described by Wang et al. [77]) have shown promise as indicators of nephropathy, particularly in diabetic populations. Spectroscopy-based markers, such as FTIR spectral bands, have also demonstrated excellent accuracy for detecting advanced CKD, with studies by Tangwanichgapong et al. [3] and Lin et al. [5] reporting AUC values of up to 1.0. These findings collectively support the development of multi-marker salivary panels for real-time monitoring, dietary guidance, and early clinical intervention.

Importantly, the reliability of salivary diagnostics can be influenced by oral health status. Pham & Le [68] observed that CKD progression is associated with deteriorating oral conditions, including reduced salivary flow and a higher Decayed, Missing, and Filled Teeth (DMFT) index, which can affect biomarker concentration and stability. Salivary composition may be altered by xerostomia, uremic halitosis, or systemic acidosis, introducing variability. Additionally, differences in collection methods, such as passive drool versus swabbing, can impact reproducibility. Given their routine patient contact and procedural standardization, dental clinics may serve as optimal environments for implementing saliva-based CKD screening, particularly when oral health assessments are included.

From a technological standpoint, a wide array of analytical methods has been used to detect CKD-related biomarkers in saliva. Electrochemical sensors, like the DPV-based system employed by Khursheed et al. [63], offer ultra-sensitive detection for creatinine. Spectroscopic techniques such as ATR-FTIR, used by Lin et al. [5] and Tangwanichgapong et al. [3], provide high-resolution molecular fingerprinting. Mass spectrometry platforms (e.g., LC-MS/MS and UPLC-MS/MS) have enabled precise detection of protein and amino acid profiles, as demonstrated by Picolo et al. [2] and Wang et al. [77]. Colorimetric assays remain prevalent, especially in resource-limited settings, while biosensing probes and salivary conductivity devices, as seen in the studies by Lu et al. [66] and Lin et al. [5], offer point-of-care potential for indirect renal function assessment.

From a clinical applicability perspective, point-of-care salivary biosensors offer rapid turnaround times (often <10 min), minimal sample volume requirements (<200 µL), and potential for integration into chairside or home-monitoring workflows [37,39]. Colorimetric test strips and dipsticks are low-cost (<USD 1 per test) and require minimal operator training, while advanced electrochemical and spectroscopic devices involve higher initial costs but can multiplex biomarker detection, potentially reducing per-test cost over time. Cost-effectiveness analyses remain scarce but are essential to guide procurement decisions, particularly in low-resource settings.

Regulatory pathways for clinical deployment will depend on jurisdiction. In the EU, biosensor devices must comply with In Vitro Diagnostic Regulation (IVDR 2017/746) requirements, including demonstration of analytical validity, clinical performance, and safety. In the US, FDA clearance under the 510(k) pathway or De Novo classification is likely required. Early engagement with regulatory bodies may accelerate translation from prototype to clinical product.

Despite these advancements, several challenges continue to impede the clinical adoption of salivary diagnostics for CKD. These include variability in collection protocols (e.g., stimulated vs. unstimulated saliva, time of day), interference from oral health conditions, and a lack of standardized cutoff values or reference ranges. Moreover, studies have not consistently accounted for population-specific biological variability, with limited representation across different racial and ethnic groups, such as Asian, African, or European populations, which may influence biomarker expression and diagnostic thresholds. The limited validation in early-stage CKD and pediatric populations, regulatory barriers for point-of-care devices, and the high cost of advanced technologies like mass spectrometry constrain broader clinical implementation.

To overcome these barriers, several key strategies should be pursued. These include developing standardized operating procedures (SOPs) for saliva collection, storage, and analysis; conducting large-scale validation studies across diverse populations (including pediatric, diabetic, and hypertensive groups); and integrating multi-analyte biosensors into wearable or chairside platforms with cloud-based data management. Additionally, incorporating oral health assessments into diagnostic workflows, establishing international calibration standards, and designing clinical trials to assess the impact of salivary monitoring on referral decisions and patient outcomes will be essential. Finally, cross-disciplinary training and collaboration among nephrologists, dentists, and primary care providers can facilitate the integration of saliva-based diagnostics into routine healthcare.

The limitations of the present scoping review include the exclusion of pediatric populations, restriction to studies published in English, and the requirement for a minimum of 20 CKD participants and a control group. This sample size threshold, determined a priori by consensus of the review team, was intended to reduce the risk of unstable or inflated diagnostic accuracy estimates—such as correlation coefficients and AUC values—that can occur in very small studies, thereby ensuring a minimum level of statistical robustness. However, this criterion may have resulted in the omission of smaller or non-comparative studies that could still offer relevant preliminary insights [100].

Although formal risk-of-bias assessment tools (e.g., QUADAS-2) are not required for scoping reviews according to the PRISMA-ScR guidelines [18], we considered potential sources of bias in the included studies. Selection bias may have occurred due to recruitment from specialized nephrology or dental clinics, potentially limiting generalizability. Reporting bias is possible, as studies with statistically significant salivary–serum correlations may be more likely to be published. Methodological heterogeneity was evident in saliva collection protocols (stimulated vs. unstimulated, fasting vs. non-fasting, swab vs. passive drool), analytical methods (colorimetry, electrochemical biosensing, ATR-FTIR, mass spectrometry), and cut-off thresholds. These differences can influence diagnostic metrics and reduce comparability, particularly when evaluating emerging biomarkers where standard protocols are not yet established. A lack of adjustment for confounding variables such as oral health status, comorbidities (e.g., diabetes), and medication use may also impact biomarker concentrations and diagnostic performance.

The heterogeneity observed across the included studies—spanning biomarker types, cut-off thresholds, saliva collection and processing methods, and analytical platforms—precluded meaningful pooling of data in a meta-analysis. Even for well-established biomarkers like creatinine and urea, the lack of standardized protocols and variable reporting of diagnostic metrics limit the statistical combination of results. This underscores the urgent need for harmonized methodologies to enable future quantitative syntheses and robust evidence-based recommendations for clinical practice.

## 5. Conclusions

This scoping review highlights the growing body of evidence supporting the diagnostic utility of saliva in the detection and monitoring of CKD. Salivary biomarkers such as creatinine and urea consistently demonstrate strong correlations with serum levels and CKD staging, with several studies reporting high diagnostic accuracy (AUCs > 0.90) and sensitivities and specificities approaching those of traditional blood and urine tests.

The review also underscores the emergence of novel salivary biomarkers—such as trimethylamine N-oxide (TMAO), cystatin variants, and specific amino acids—which may enhance early-stage detection and disease stratification, particularly when integrated into multi-analyte panels.

Importantly, advances in biosensing technologies, including electrochemical sensors, ATR-FTIR spectroscopy, and portable point-of-care devices, offer promising solutions to current diagnostic limitations by enabling rapid, non-invasive, and decentralized monitoring. However, challenges such as variability in saliva composition, oral health influence, lack of standardized collection protocols, and limited validation in early CKD stages must be addressed before widespread clinical implementation.

To translate salivary diagnostics into routine clinical and dental workflows, future research should prioritize:

- development and adoption of standardized operating procedures (SOPs) for saliva collection, storage, and biomarker analysis;
- large-scale, multicenter validation studies across diverse populations (including early-stage CKD, diabetic, and hypertensive patients);
- integration of oral health assessments into diagnostic algorithms;
- comprehensive health economic analyses to determine cost-effectiveness compared to conventional testing; and
- regulatory approval and post-market surveillance of biosensor devices.

Interdisciplinary collaboration between nephrology, dentistry, biomedical engineering, and health economics will be essential to advance saliva as a viable alternative to blood and urine in CKD care.

## Figures and Tables

**Table 1:** Research question formulated based the PICOS framework.

Component	Description
**P (Population)**	Adults (≥18 years) diagnosed with chronic kidney disease (any stage) or individuals at risk of CKD (e.g., with diabetes, hypertension, cardiovascular disease)
**I (Intervention/Exposure)**	Use of salivary biomarkers (e.g., urea, creatinine, ammonia, pH, uric acid, cystatin C) for the detection or monitoring of CKD, including application of digital diagnostic tools such as biosensors or lab-on-a-chip technologies
**C (Comparator)**	Traditional blood- and urine-based diagnostic methods (e.g., serum creatinine, eGFR, urinary albumin-to-creatinine ratio, 24 h creatinine clearance)
**O (Outcomes)**	Diagnostic accuracy metrics (sensitivity, specificity, predictive values, correlation coefficients, AUC); feasibility and clinical utility of salivary diagnostics
**S (Study Design)**	Observational studies (cross-sectional, case–control, cohort), diagnostic accuracy studies, and clinical validation studies involving human participants

**Table 2:** Characteristics of the included studies.

Author, Year, Location, Setting	Study Design	Participants (CKD/Control)	Biomarkers Investigated	Collection & Analysis Methods	Key Findings & Outcomes	Diagnostic Accuracy
Khursheed et al., 2025, Pakistan, University [63]	Cross-sectional	27 total (saliva, serum, urine from patients with high/low creatinine, 9 controls)	Creatinine	Electrochemical detection via DPV with Ag@GO/TiO2-GCE sensor	Saliva creatinine recovery 91–97%; superior to Jaffe’s method	Sensitivity: 15.74 µA/pM.cm^2^, LOD: 1.15 pM, AUC not reported
Picolo et al., 2025, Brazil, University [2]	Pilot cross-sectional	10 ESKD, 10 controls	Proteomic markers (API5, PI-PLC, Sgsm2)	LC-MS/MS, amylase depletion	3 proteins absent in CKD, present in controls	AUC: ~0.8, suggested biomarker potential
Tangwanichgapong et al., 2025, Thailand, University [3]	Cross-sectional matched-pair	24 ESKD, 24 controls	Salivary spectral bands	ATR-FTIR spectroscopy	Clear biochemical spectral differences between ESKD and controls	Accuracy: 87.5–100%, Sensitivity: 75–100%, Specificity: 100%
Choudhry et al., 2024, India, University [61]	Cross-sectional	30 CKD, 30 controls	Urea, Creatinine	Passive drool, autoanalyzer	Significant group difference; strong correlations	Urea AUC: 0.78, Sensitivity: 90%, Creatinine AUC: 0.86, Sensitivity: 89%
Ashwini et al., 2023, India, Hospital [82]	Cross-sectional	20 CKD (stages 3–5), 20 controls	Creatinine	Spitting after fasting; Jaffe’s method	Strong serum/saliva correlation	AUC: 0.879, Sensitivity: 75%, Specificity: 90%
Korytowska-Przybylska et al., 2023, Poland, University [64]	Observational	31 CKD, 20 controls	TMAO, Creatinine	Salivette swab, LC-MS/MS	TMAO more effective for stage IV discrimination	No AUC; correlation with CKD stage
Nagarathinam et al., 2023, India, Hospital [79]	Cross-sectional	150 CKD across 5 stages/30 controls	Urea	Unstimulated saliva; spitting; GLDH enzymatic assay	Salivary urea progressively increased across CKD stages	AUC: 0.917; Sensitivity: 88%, Specificity: 84%, Cutoff: 28.25 mg/dL
Pillai et al., 2023, India, Dental Hospital [69]	Case–control	120 total (30 controls, 90 CKD stage 3–5)	Urea, Creatinine	Spit technique, centrifuge, colorimetry	Significant correlation between saliva and serum	No diagnostic metrics
Poposki et al., 2023, N. Macedonia, University [70]	Cross-sectional	32 CKD (stages 2–5), 20 controls	Urea, Creatinine, Albumin, Uric acid	Unstimulated saliva, centrifuge	Salivary urea correlated with CKD stage	No AUC; correlation stats given
Shamsan et al., 2023, Yemen, Sana’a University [71]	Cross-sectional	59 renal disease patients/20 controls	Multiple electrolytes, Creatinine, Urea, TP, Albumin	Unstimulated saliva; colorimetry via Chemray 240	Elevated renal biomarkers across all saliva samples	No diagnostic metrics; statistically significant
Wang et al., 2023, China, University [77]	Observational	90 total (30 DN,30 Type II DM, 30 controls)	Amino acids (arginine, valine, histidine)	UPLC-MS/MS	Combined biomarker model highly predictive	Combined AUC: 0.957, Saliva Arginine AUC: 0.75
Lin et al., 2022, Taiwan, Hospital [5]	Pilot cross-sectional	214 adults, CKD prevalence 11.2%	Conductivity (indirect biomarkers)	Swab collection + biosensing probe	Conductivity correlates with CKD indicators	AUC: 0.648 (conductivity alone), 0.798 with age/gender/weight
Lin et al., 2022, UK, University College London [74]	Diagnostic accuracy	20 CKD (stages 1–5), 6 controls	Urea	ATR-FTIR spectroscopy	Significant differentiation by stage	AUC: up to 1.00 (CKD 4–5), Sensitivity: 100%, Specificity: up to 100%
Padwal et al., 2022, India, Hospital [67]	Case–control	50 CKD (stages 4–5), 50 controls	Creatinine, Urea	Spitting method, enzymatic and Jaffe’s methods	Significant elevation in CKD; strong correlations	Creatinine AUC: 1.000, Sensitivity/Specificity: 100%; Urea AUC: 0.98
Trzcionka et al., 2021, Poland, University [73]	Observational	180 CKD on dialysis, 48 controls	Saliva flow, pH, buffering	Saliva-Check buffer kit	Hemodialysis reduces flow, alters buffer	No diagnostic metrics
Harish et al., 2020, India, University [62]	Observational	180 total (90 controls, 90 diabetics ± nephropathy)	Urea, Creatinine, Glucose, Uric acid	Fasting, spitting, centrifuge, autoanalyzer	CKD group shows elevated levels; saliva tracks serum well	No AUC reported; significant correlations
Lu et al., 2019, Taiwan, University [66]	Clinical validation	30 total (10 CKD, 10 healthy adults, 10 farmers)	Saliva conductivity	Swab collection, Au electrode sensing	Significant differences across groups	Sensitivity: 93%, Specificity: 80%
Pham & Le, et al., 2019, Vietnam, Hospital [68]	Cross-sectional	111 CKD, 109 non-CKD	Urea, Creatinine, Flow rate	Dual saliva collection, chem analyzer	Xerostomia & DMFT worsen with CKD stage	Regression R^2^ for flow rate: 0.75
Techatanawat et al., 2019, Thailand, Hospital [72]	Observa tional	82 subjects (29 DM, 20 DN, 8 NDIN, 25 controls)	Cystatin SA	ELISA, proteomics	Cystatin SA tracks nephropathy severity	Salivary levels showed upward trends; no AUC reported
Yan et al., 2019, China, University [78]	Observational	27 CKD/27 controls	L-phenylalanine, L-tryptophan, Creatinine	LC-MS/MS with hydrophilic chromatography	Salivary levels elevated in CKD; significant correlation	Combined AUC: 0.936, Sensitivity: 88.9%, Specificity: 92.6%
Alsamarai et al., 2018, Iraq, University [81]	Case–control	29 CKD, 20 controls	Cystatin C, Urea, Creatinine	ELISA, colorimetric methods	Cystatin C shown as superior saliva marker	No AUC reported
Bilancio et al., 2018, Italy, University [85]	Observational	30 CKD, 15 controls	Phosphorus, Urea	Salivette method, molybdate UV, NADH methods	Saliva correlates highly with plasma; reproducible method	No diagnostic metrics; strong correlations reported
Pham et al., 2017, Vietnam, University [76]	Diagnostic study	112 CKD, 108 controls	Urea, Creatinine	Spitting after fasting, analyzer	CKD group had elevated levels; strong correlation	Creatinine AUC: 0.92, Sensitivity: 86.5%, Specificity: 87.2%
Bagalad et al., 2016, India, University [84]	Case–control	41 CKD, 41 controls	Urea, Creatinine, Electrolytes	Spit method, centrifuge, autoanalyzer	All CKD biomarkers elevated; cutoff values established	Creatinine AUC: 0.90, Sensitivity: 93%, Specificity: 90%
Lasisi et al., 2016, Nigeria, University [9]	Cross-sectional	50 CKD (stages 4–5), 49 controls	Urea, Creatinine	Unstimulated whole saliva; Jaffe & Marsh methods	Salivary levels significantly elevated; strong correlation with serum	Creatinine AUC: 0.97, Sensitivity: 94%, Specificity: 85%
Abeer Hamdy, et al., 2015, Egypt, University [83]	Cross-sectional	40 CKD (incl. ESKD)/10 healthy controls	Urea, Creatinine	Unstimulated saliva; passive drool; colorimetric and rate techniques	Significant serum–saliva correlation across CKD stages	Creatinine AUC: 0.876; Sensitivity: 92%, Urea AUC: 0.796; Sensitivity: 90%
Venkatapathy et al., 2014, India, University [75]	Case–control	105 CKD (stage 4/5), 37 controls	Creatinine	Spitting technique; autoanalyzer; Jaffe method	Salivary creatinine elevated; strong correlation with serum	AUC: 0.967; Sensitivity: 97.14%, Specificity: 86.5%; Cutoff: 0.2 mg/dL
Lloyd et al., 1996, UK, Hospital [65]	Diagnostic accuracy	26 CKD/23 healthy	Creatinine	Stimulated mixed saliva; chewing gum; Jaffe rate reaction	Salivary creatinine significantly elevated; strong CKD-specific correlation	Sensitivity: up to 100%, Specificity: up to 100%, AUC: ~0.97
Akai et al., 1983, Japan, University [80]	Method validation	44 CKD/12 controls	Urea nitrogen	Dry-reagent test strip; reflectance spectrometer	High correlation (r = 0.93) with serum levels; method simple and reliable	No AUC; r values indicate diagnostic potential

DPV = Differential Pulse Voltammetry, AUC = Area Under the Curve, LOD = Limit of Detection, Ag@GO = Silver nanoparticles (Ag) integrated with Graphene Oxide (GO), GCE = Glassy Carbon Electrode, ESKD = End-Stage Kidney Disease, API5 = Apoptosis Inhibitor 5, PI-PLC = Phosphatidylinositol-specific Phospholipase C, Sgsm2 = Small G Protein Signaling Modulator 2, LC-MS/MS = Liquid Chromatography–Tandem Mass Spectrometry, CKD = Chronic Kidney disease, ATR-FTIR spectroscopy = Attenuated Total Reflectance Fourier Transform Infrared Spectroscopy, TMAO = Trimethylamine N-oxide, GLDH = Glutamate Dehydrogenase, UPLC-MS/MS = Ultra Performance Liquid Chromatography Tandem Mass Spectrometry, DMFT = Decayed, Missing, and Filled Teeth, DM = Diabetes Mellitus, DN = Diabetic Nephropathy, NDIN = Non-Diabetic Individuals with Nephropathy, ELISA = Enzyme-Linked Immunosorbent Assay, NADH Method = Nicotinamide Adenine Dinucleotide—Hydrogen Method.

**Table 3:** Diagnostic Performance of Salivary Creatinine and Urea Across Studies.

Biomarker	Study	AUC	Sensitivity/Specificity	Additional Observations
Creatinine (2-Amino-1-methyl-5H-imidazol-4-one) 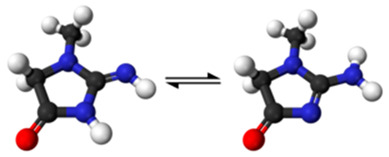	Padwal et al., 2022 [67]	1.000	100%/100%	Excellent accuracy using enzymatic and Jaffe’s methods
	Venkatapathy et al., 2014 [75]	0.967	97.14%/86.5%	Strong serum correlation; cutoff: 0.2 mg/dL
	Lasisi et al., 2016 [9]	0.970	94%/85%	Strong correlation with serum
	Pham et al., 2017 [76]	0.920	86.5%/87.2%	Based on fasting samples
	Bagalad et al., 2016 [84]	0.900	93%/90%	Cutoff values established
	Abeer Hamdy et al., 2015 [83]	0.876	92%/not reported	Good correlation with CKD stage
	Ashwini et al., 2023 [82]	0.879	75%/90%	Good serum correlation; Jaffe’s method used
	Choudhry et al., 2024 [61]	0.860	89%/not reported	Passive drool method
	Khursheed et al., 2025 [63]	Not reported	Sensitivity: 15.74 µA/pM.cm^2^	Electrochemical detection; strong recovery rates
Urea (Carbonic diamide) 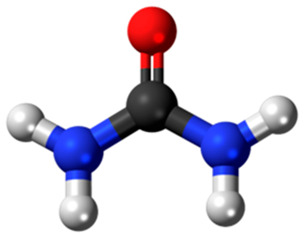	Padwal et al., 2022 [67]	0.980	Not specified	Colorimetric method
	Nagarathinam et al., 2023 [79]	0.917	88%/84%	Clear stage-wise increase; GLDH enzymatic assay
	Abeer Hamdy et al., 2015 [83]	0.796	90%/not reported	Passive drool technique
	Choudhry et al., 2024 [61]	0.780	90%/not reported	Saliva/serum correlation-strong
	Ashwini et al., 2023 [82]	Not reported	75%/90%	Spitting technique after fasting

Creatinine and urea symbols retrieved from Wikimedia Commons (CC0 license).

**Table 4:** Emerging or Novel Biomarkers.

Biomarker	Diagnostic Potential	Study/Additional Observations
TMAO	Correlated with stage IV	Korytowska et al., 2023 [64]/may help in stage-specific detection
Cystatin (SA, C)	Trend correlates with severity	Techatanawat et al., 2019 [72]; Alsamarai et al., 2018 [81]
Proteins (API5, PI-PLC, Sgsm2)	Present in controls, absent in CKD	Picolo et al., 2025 [2]/AUC ~0.8
L-phenylalanine & L-tryptophan	Combined AUC = 0.936	Yan et al., 2019 [78]
Conductivity	AUC: 0.648 (alone), 0.798 with demographics	Lin et al., 2022 [5]; Lu et al., 2019 [66]/showed 93% sensitivity
pH	Average salivary pH was: Higher in the control group (~7.0) Lower in CKD patients, especially those with diabetes (e.g., 5.96 in CKD + diabetes group)	Trzcionka et al., 2021 [73]/pH was not directly used as a diagnostic marker, but is an indirect indicator of salivary alterations in CKD, particularly in advanced stages/comorbid conditions.

## Data Availability

The original contributions presented in this study are included in the article. Further inquiries can be directed to the corresponding author.

## Abbreviations

The following abbreviations are used in this manuscript:

CKD	Chronic Kidney disease
eGFR	estimated Glomerular Filtration Rate
KDIGO	Kidney Disease: Improving Global Outcomes
ACR	Albumin-to-Creatinine Ratio
AUC	Area Under the Curve
ESKD	End-Stage Kidney Disease
DPV	Differential Pulse Voltammetry
LOD	Limit of Detection
Ag@GO	Silver nanoparticles (Ag) integrated with Graphene Oxide (GO),
GCE	Glassy Carbon Electrode
API5	Apoptosis Inhibitor 5
PI-PLC	Phosphatidylinositol-specific Phospholipase C
LC-MS/MS	Liquid Chromatography–Tandem Mass Spectrometry
ATR-FTIR spectroscopy	Attenuated Total Reflectance Fourier Transform Infrared Spectroscopy
TMAO	Trimethylamine N-oxide
PICOS	Population, Intervention, Comparator, Outcome, Study Design
PRISMA-ScR	Preferred Reporting Items for Systematic Reviews and Meta-Analyses for scoping reviews
sCR	Serum creatinine
BUN	Blood urea nitrogen
DMFT	Decayed, Missing, and Filled Teeth
UPLC-MS/MS	Ultra Performance Liquid Chromatography -Tandem Mass Spectrometry
SOPs	Standardized operating procedures
